# Transcription factor networks in trophoblast development

**DOI:** 10.1007/s00018-022-04363-6

**Published:** 2022-06-03

**Authors:** Henrieta Papuchova, Paulina A. Latos

**Affiliations:** Center for Anatomy and Cell Biology, Medical University of Vienna, Schwarzspanierstrasse 17, 1090 Vienna, Austria

**Keywords:** Trophoblast, Transcription factors, Human placenta, Human trophoblast stem cells

## Abstract

The placenta sustains embryonic development and is critical for a successful pregnancy outcome. It provides the site of exchange between the mother and the embryo, has immunological functions and is a vital endocrine organ. To perform these diverse roles, the placenta comprises highly specialized trophoblast cell types, including syncytiotrophoblast and extravillous trophoblast. The coordinated actions of transcription factors (TFs) regulate their emergence during development, subsequent specialization, and identity. These TFs integrate diverse signaling cues, form TF networks, associate with chromatin remodeling and modifying factors, and collectively determine the cell type-specific characteristics. Here, we summarize the general properties of TFs, provide an overview of TFs involved in the development and function of the human trophoblast, and address similarities and differences to their murine orthologs. In addition, we discuss how the recent establishment of human in vitro models combined with -omics approaches propel our knowledge and transform the human trophoblast field.

## Introduction

The placenta is the most diverse organ among mammals, and it aids fetal development by facilitating nutrient, metabolite and gas exchange between the mother and the fetus. To fulfill these various functions, the progenitor cells of the placenta differentiate into multiple highly specialized trophoblast cell types. Proper trophoblast differentiation is essential for correct placental and fetal development as aberrant differentiation often results in pregnancy complications, hampering both maternal and fetal health. This review focuses on trophoblast cell identity determined by specific gene expression patterns driven by transcription factors (TFs). They form networks, and together with co-factors, chromatin-modifying and -remodeling complexes govern cell identity and differentiation. The review is divided into three main parts describing (i) the development of the human placenta, (ii) general properties of TFs and (iii) human trophoblast cell identity determined by TFs, where we discuss individual TFs and signaling pathways in trophectoderm (TE), cytotrophoblast (CTB), syncytiotrophoblast (STB) and extravillous trophoblast (EVT) in comparison to mouse placenta development.

### Development of the human placenta

**Fig. 1:**
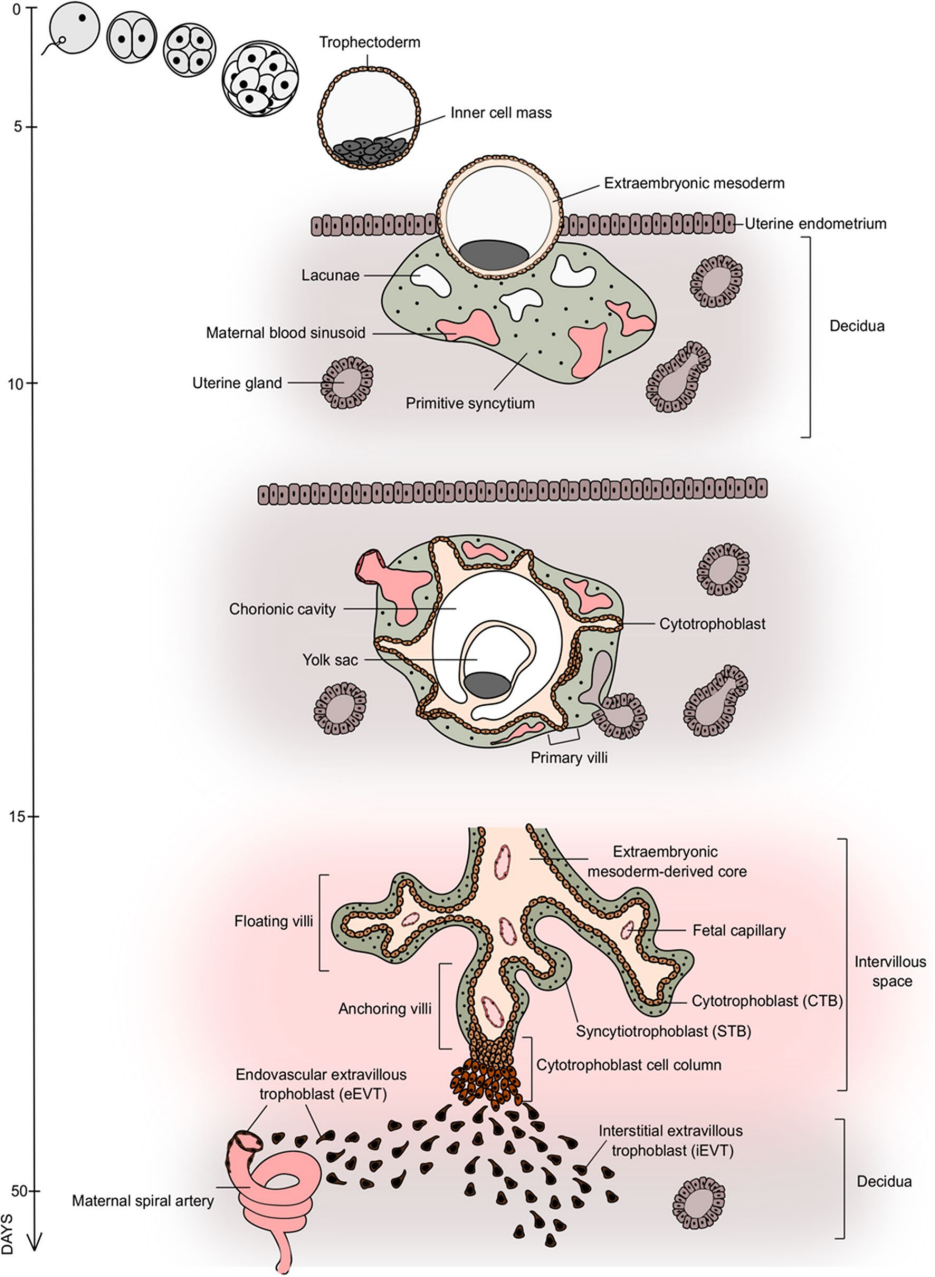
The development of the human placenta. Initiated by fertilization, the zygote undergoes multiple divisions and gives rise to a blastocyst 4–5 days post-fertilization (dpf). The blastocyst consists of the inner cell mass, which gives rise to the embryo proper and the trophectoderm, which gives rise to the trophoblast of the placenta. Upon implantation of the blastocyst into the uterine endometrium, the establishment of the multi-nucleated primitive syncytium (PS) and the cytotrophoblast (CTB) monolayer begins. Around day 9 dpf, lacunae form within the PS, fuse subsequently with uterine capillaries, and establish maternal sinusoids filled with maternal blood by day 13 dpf. Simultaneously, the CTB expands through the PS, extending towards the maternal decidua and forming primary villi. By the end of the third trimester, the main placental structure, the villous tree, is fully established. The villous tree consists of the extraembryonic mesoderm-derived core, fetal capillaries, the CTB monolayer and the multinucleated syncytiotrophoblast (STB) layer, arising from CTB fusion. The floating villi of the villous tree are located in the intervillous space filled with maternal blood, while the anchoring villi extend towards the decidua. At the tip of the anchoring villi, CTB forms a cytotrophoblast cell column, comprising the proliferative progenitor population of invasive extravillous trophoblast (EVT). The EVT can be divided into two subtypes; the endovascular EVT (eEVT), which remodels the maternal spiral arteries and the interstitial EVT (iEVT), which invades the decidua

**Fig. 2:**
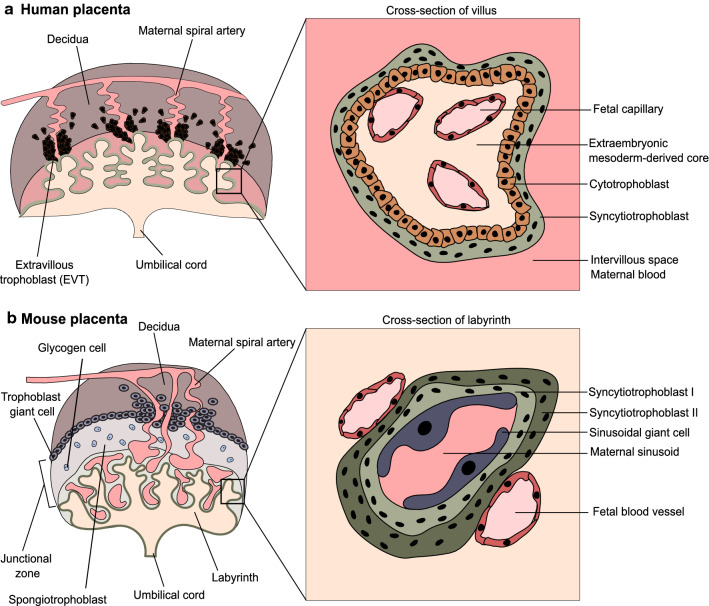
Comparison of the human and murine placenta. **a** The main unit of the human placenta is a villous tree, comprising the extraembryonic mesoderm-derived core (ExM-dC), containing fetal capillaries. The ExM-dC is covered by the cytotrophoblast (CTB) monolayer and the multinucleated syncytiotrophoblast (STB) layer that is in contact with maternal blood. The inset shows a cross-section through the villi. At the tip of the villi, the cytotrophoblast cell column forms. It gives rise to the interstitial extravillous trophoblast (iEVT), invading the decidua and the endovascular extravillous trophoblast (eEVT), invading the maternal spiral arteries. **b** The murine placenta can be divided into the junctional zone and the labyrinth zone. The junctional zone consists of the spongiotrophoblasts and the glycogen cells. The trophoblast giant cells invade the maternal decidua and remodel maternal arteries and thus are considered to correspond to human EVT. In the labyrinth zone, the fetal blood is separated from the maternal blood sinusoids by fetal endothelial cells, two layers of STB (STB-I and STB-II) and sinusoidal giant cells. The labyrinth functionally relates to human villi

During human development, the first lineage decision segregates the TE and the inner cell mass (ICM), resulting in the blastocyst 4–5 days post fertilization (dpf). While all embryonic cells originate from the ICM, the TE generates the trophoblast compartment of the placenta. The blastocyst implants into the maternal endometrium 6–7 dpf; the TE gives rise to the mononuclear CTB that differentiates into the invasive multinuclear primitive syncytium (PS) (Fig. 1) [1]. Around day 9, vacuoles appear in the PS that fuse and form the lacunar spaces, and eventually breach the maternal capillaries giving rise to the maternal blood sinusoids [2]. Concomitantly, rows of proliferative CTB break through the expanding PS and form primary villi, subsequently invaded by the extraembryonic mesoderm (ExM), which is thought to originate from the ICM. Further proliferation and differentiation result in a villous structure consisting of an ExM-derived core containing fetal capillaries covered by two trophoblast layers: the proliferative villous CTB and its derivative, the multinuclear STB (Fig. 1) [1]. The STB forms a syncytium that provides the exchange site between the maternal and fetal bloodstreams and produces and secretes a plethora of pregnancy hormones. The developing placenta comprises two types of villi: the floating villi and the anchoring villi attached to the decidua through cell columns (Fig. 1). The proximal part of the column is a proliferative progenitor pool that gives rise to the highly invasive and migratory EVT lineage. Two distinct EVT populations exist by 15–16 dpf and safeguard immunological adaptation: the interstitial EVT (iEVT) that invade decidual stroma and the endovascular EVT (eEVT) that colonize and remodel maternal spiral arteries to a high conductance at low-pressure vessels, ensuring optimal blood flow [3]. In addition to the arterial transformation, the eEVT moves down the artery and forms plugs that prevent blood flow and safeguard a hypoxic environment. By the end of the first trimester, the plugs disintegrate, and the three main trophoblast lineages of the human placenta are established: the CTB, the STB and the EVT, providing an outline for further growth and physiological development (Figs. 1 and 2a).

While human and murine placentas fulfill analogous functions and both show a hemochorial (maternal blood directly contacts trophoblast cells) type of placentation, they exhibit several morphological and functional differences. The murine placenta comprises the junctional and the labyrinth zones (Fig. 2b). The junctional zone consists of cells derived from the ectoplacental cone (EPC), encompassing the secondary trophoblast giant cells (TGCs), the spongiotrophoblast cells, and the glycogen cells (GlycCs) [4]. The secondary TGCs are thought to correspond to EVTs, as they invade (around E7.5-E9.5) deeply into the decidua and remodel the maternal spiral arteries. The GlycCs are loaded with glycogen and serve as an energy store. The labyrinth zone provides the site of the maternal–fetal exchange and functionally relates to human chorionic villi. During murine development, the TE gives rise to the extraembryonic ectoderm (ExE) and the EPC. The allantois arises from the ExM, and around embryonic day E8.5 fuses with the chorion, a structure of ExE origin, in a process known as the chorioallantoic fusion [5]. This fusion enables the invasion of the ExM-derived blood vessels into the chorionic layer, eventually leading to the establishment of the labyrinth zone. Here, the fetal and the maternal bloodstreams are separated by a trilaminar barrier consisting of the two layers of syncytiotrophoblast and a layer of sinusoidal giant cells lining the maternal blood sinuses [6] (Fig. 2b). Thus, the general functional layout of the murine and human placenta is similar, as in both cases, the fetal vasculature is of ExM origin, and the maternal blood flows in trophoblast-lined sinuses and remains in direct contact with STB; however, species-specific differences do exist. Despite these disparities, the murine placenta has proven to be a very informative animal model, not least due to its amenability to genetic engineering, particularly gene knockout (KO) experiments [7, 8]. In addition, derivation of self-renewing and multipotent mouse trophoblast stem cells (mTSCs), representing the ExE compartment of the mouse embryo [9], contributed enormously to our understanding of the TF networks operating in trophoblast.

**Fig. 3:**
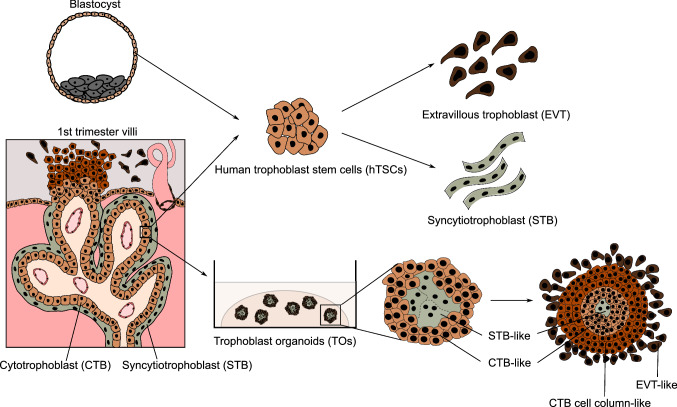
Human trophoblast stem cells and trophoblast organoids. The human trophoblast stem cells (hTSC) can be derived from both the blastocyst and the cytotrophoblast (CTB) of the first-trimester placenta. The hTSCs represent the proliferative CTB population and can be differentiated into extravillous trophoblast (EVT) and syncytiotrophoblast (STB) upon defined culture conditions. Moreover, trophoblast organoids (TOs) can be established from the first-trimester CTB. TOs have an STB-like core and CTB-like shell and thus provide an inside-out three-dimensional model. TOs can be further differentiated to form the cytotrophoblast cell column (CCC)-like and EVT-like populations

Numerous human placental disorders have their pathophysiological roots at the early developmental stages. However, molecular studies of the underlying causes were long hampered by the inaccessibility of placental tissue and the lack of suitable in vitro models. Researchers mainly relied on the scarcely available primary tissue and several transformed and cancer cell lines of restricted potential [5]. While animal models, in particular mice, are widely used to study the development and function of human organs, their ability to model the placenta is limited. As described above, the development of the specific trophoblast lineages and cell types differ between the two species. For instance, various types of the trophoblast giant cells feature prominently in the murine placenta while these are limited to EVT subtypes in humans [4, 10]. The recent establishment of the human trophoblast stem cells (hTSCs) and self-organizing trophoblast organoids (TOs) was a major breakthrough and provided novel, reliable tools to study molecular mechanisms driving trophoblast development and disease (Fig. 3) [11–13]. The hTSCs represent the CTB population of the first trimester placenta. In culture conditions activating EGF and WNT and inhibiting TGF-β and ROCK signaling, they self-renew and remain bipotent. Upon induction, hTSCs can differentiate into STB or EVT, providing a versatile in vitro system to follow trophoblast development [11]. TOs require similar signaling inputs to self-organize in 3D into the outer CTB-like layer and the inner STB-like compartment, representing the inside-out model of the human villi [12, 13]. While these models have already proven to be a powerful tool to interrogate TF function in CTB [14–16], naive human embryonic stem cells (hESCs) conversion into hTSCs and recently-derived human blastoids can be used to dissect even earlier stages of human trophoblast development [17–24].

### General properties of TFs controlling gene regulatory networks

Cell identity is determined by the specific TF-driven transcriptional outputs and is coordinated by the signaling inputs [25]. TFs bind DNA in a sequence-specific manner, cooperate with chromatin remodeling and modifying complexes, and other factors to recruit the transcriptional machinery and drive transcription. Specific cell types are usually defined by a particular combination of TFs, as evidenced by cell (re)programming studies. Classical reprogramming experiments demonstrated that transient ectopic expression of *Oct4*, *Sox2*, *Klf4* and *Myc* (OSKM) in fibroblasts cultured in murine ESC media resulted in the establishment of self-renewing induced pluripotent stem cells (iPSCs), barely distinguishable from ESCs [26]. Similarly, transient, ectopic expression of *Eomes*, *Tfap2c*, *Gata3* and optionally *Ets2* or *Myc* in mouse fibroblast coupled to mouse TSC culture conditions (FGF4 and TGF-β) led to the establishment of self-renewing and multipotent induced TSCs (iTSCs) that contributed to the placenta in chimeras [27, 28]. Moreover, a recent report showed reprogramming of human term CTB to iTSCs upon expression of 5 TFs (*TFAP2C*, *TEAD4*, *CDX2*, *ELF5* and *ETS2*) in hTSC culture conditions [29]. The role of TFs in translating the signaling inputs into the transcriptional outputs was demonstrated in the trophoblast context, as constitutive overexpression of *Sox2* and *Esrrb*/*Tfap2c* was sufficient to confer FGF4-independent self-renewal and sustain multipotency in mTSCs [30].

Importantly, TFs usually do not function as a sum of single players but rather operate as networks at both the transcriptional and protein level [31–35]. Analysis of TFs in mTSCs (e.g., ESRRB, TFAP2C, TEAD4, GATA2/3, SOX2, ELF5, EOMES, CDX2, etc.) revealed that they directly bind their own and each other's genes and drive their expression resulting in the maintenance of the network [30, 36–38]. For instance, ESRRB binds itself as well as the *Eomes* and *Elf5* genes and is essential for their expression [36]. Furthermore, in addition to sustaining each other, trophoblast TFs co-bind and co-regulate the expression of downstream gene modules determining the mTSC identity [38]. The co-binding preferentially occurs at the gene regulatory regions referred to as enhancers. Enhancers are densely packed with DNA motifs bound by TFs, are highly enriched for transcriptional cofactors (e.g., p300), exhibit accessible chromatin structure and may cooperate and form regulatory hubs referred to as super-enhancers [39]. A recent study in differentiating mTSCs systematically mapped trophoblast-specific super-enhancers and the associated TFs, of which the vast majority has not been characterized yet in the placental context [38]. Interestingly, no bonafide trophoblast-specific TFs have been identified, as all of the known essential trophoblast TFs are also expressed in a range of embryonic and adult cell types, indicating that cell-type specificity is acquired by their unique combinations. This notion is exemplified by EOMES, CDX2 and SOX2 that function largely separately in the early mesendoderm, late mesoderm, and neuroectoderm lineage derivatives, respectively, but uniquely cooperate in the trophoblast lineage [40–43]. Another example is provided by the key stem cell/progenitor TF SOX2 that has context-dependent protein interactors and therefore different target gene networks (e.g., OCT4 in mESC, TFAP2C in trophoblast, and OTX2 in neuroectoderm) [30, 34, 44–46]. The acquisition of new TF combinations in a network could be driven by the evolution of novel cis-regulatory elements, in agreement with the genetic theory of morphological evolution [47]. It has been reported that endogenous retroviruses function as species-specific placental enhancers bound by CDX2, EOMES and ELF5 TFs [48]. The tissue-specificity of these enhancers relies on the low methylation levels in trophoblast, providing access to otherwise silenced regions (by DNA methylation), which are the source of regulatory variation [48]. Another example is a human long terminal repeat (LTR) element driving placental expression of the corticotropin-releasing hormone (CRH) via the TF DLX3 and thereby regulating gestational length. This LTR element was sufficient to reignite *CRH* expression in transgenic mice, resulting in prolonged gestational length [49]. In sum, TFs operate as a highly intertwined transcriptional regulatory circuitry, determining trophoblast cell identity.

### TF protein networks in trophoblast

TFs also form networks at the protein level. For instance, interactions of MSX2 with both GATA3 and TFAP2C [15] were identified in hTSCs, and mutual interactions for ELF5, TFAP2C and EOMES [37] as well as TFAP2C and SOX2 [30] were reported in mTSCs. Interestingly, the relative abundance of EOMES, ELF5 and TFAP2C determines the outcomes of their interactions. In mTSCs, ELF5 interacts with EOMES and recruits TFAP2C to triply occupied sites at trophoblast-specific genes, driving their expression. In contrast, the interaction of ELF5 and TFAP2C becomes predominant as their protein levels increase. This triggers binding to double- and single-occupancy sites that harbor the cognate Tfap2c motif, causing activation of the associated differentiation-promoting genes [37]. Thus, mouse trophoblast cell identity shifts in line with the stoichiometry of these associations, indicating that specificity is not determined by a single TF but by its interactions. This is further illustrated by the largely cell-type-specific protein interactomes and gene binding patterns of SOX2 and ESRRB in mTSCs vs. mESCs [30].

TFs exert their function in cooperation with transcriptional cofactors, chromatin remodeling complexes and chromatin-modifying enzymes. Transcriptional cofactors provide the link to the general RNA Polymerase II (PolII) machinery and comprise a diverse array of proteins and complexes. For instance, YAP is a TEAD4 cofactor in mTSCs and hTSCs [50–52], while the Integrator complex was reported to interact with ESRRB in mTSCs. ESRRB also interacted with components of the Lysine-specific histone demethylase 1 (LSD1), and Nucleosome Remodeling and Deacetylase (NuRD) complexes [36]. We have also recently reported a strong interaction of the TF MSX2 with the canonical BRG1/BRM associated factors (cBAF) complex, a subtype of the SWItch/Sucrose Non-Fermentable (SWI/SNF) chromatin remodeling complex, in hTSCs. Chromatin immunoprecipitation followed by high-throughput sequencing (ChIP-seq) analyses have demonstrated a substantial binding overlap between MSX2, cBAF components ARID1A and BRG1, and H3K27ac in hTSCs. Intriguingly, depletion of MSX2 resulted in spontaneous STB differentiation, increased cBAF occupancy and elevated levels of H3K27ac. Thus, in hTSCs, MSX2 keeps cBAF “in check”, prevents premature activation of STB genes and reinforces the hTSC identity [15]. These findings provide a paradigm of how the intrinsic connections between TFs, chromatin remodelers and modifiers coordinately determine transcriptional cell fate. In addition to cBAF, also non-canonical (nc)BAF and Polybromo-associated (P)BAF complexes have been shown to operate in hTSCs [15]; however, their precise functions await elucidation. The SWI/SNF complex has also been implicated in the regulation of self-renewal in mTSCs. Its core subunit BRG1 has been shown to cooperate with EOMES and TFAP2C TFs and positively regulate the expression of crucial stemness markers, including *Elf5*, *Eomes* and *Cdx2* [53]. The SWI/SNF complex is also essential for early trophoblast development as genetic ablation of *Brg1*, *Baf47*, and *Baf155* subunits resulted in developmental arrest around the blastocyst stage and implantation failure [54–56]. Other chromatin regulators involved in trophoblast self-renewal are the chromatin organizers SATB homeobox 1 (SATB1) and SATB2. Their depletion led to a loss of self-renewal and differentiation of rat TSCs [57]. In contrast, the bromodomain BPTF nucleosome remodeling factor NURF301 is necessary for trophoblast differentiation. Its gene KO resulted in diminished expression of *Ascl2* and *Hand1*, reduced/absent ectoplacental cone, and embryonic lethality [58].

Besides the chromatin remodeling status, active (e.g., H3K4me4, H3K27ac, H3K9ac) and repressive (e.g., H3K27me3, H3K9me2/3) histone modifications have been implicated in transcriptional regulation of trophoblast cell states. Genetic ablation of histone-modifying enzymes, including EZH2 (H3K27 methyltransferase), G9A (H3K9 methyltransferase), KDM6 (H3K27 demethylase), LSD1 (H3K4 demethylase), MYST1/MYST2 (H3K14 acetyltransferase) and SUV39H1 (H3K9 methyltransferase) led to severely impaired trophoblast development and embryonic lethality (reviewed in [59]). In contrast to murine, the epigenome of the human trophoblast remains relatively poorly characterized. Recently, Kwak et al. provided some new insights through genome-wide characterization of histone modifications (H3K4me3, H3K9Ac, H3K27Ac and H3K27me3) and RNA Pol II occupancy in CTB and in vitro differentiated STB. These analyses revealed that the transition from CTB to STB is associated with profound gene regulatory and epigenetic changes [60]. Accordingly, pharmacological inhibition and genetic depletion of histone deacetylase 1 and 2 impaired STB differentiation [61].

Together, these examples illustrate how TFs, chromatin remodelers, and modifiers cooperate and coordinately regulate specific transcriptional outputs that determine cell identity. They also highlight the importance of the holistic approach when studying transcriptional regulation of cell fate decisions.

### TFs determining trophoblast identity

#### TFs operating in TE

Human development commences with the fertilization and formation of a zygote. Following several cell divisions, the first lineage specification takes place at the morula stage, with the outer, polar cells giving rise to TE and the inner, apolar cells becoming the ICM. The outer, polar cells, due to the presence of a “free” apical surface, show different localization of aPKC, AMOT, and PARD6B and divergent distribution of the adherens junctions in comparison to the inner cells [52]. As a consequence of this polarization and localized mechanical strain, the HIPPO signaling pathway is differentially activated. In the inner cells, the HIPPO signaling effector YAP1 is phosphorylated and retained in the cytoplasm. In the outer cells, the unphosphorylated YAP1 translocates to the nucleus where, as a cofactor, together with TEAD4 and GATA3 activates the expression of key downstream TFs. As the HIPPO/TEAD4 pathway is required for the expression of *Gata3* in the early mouse embryo, it is likely that a similar mechanism operates in the human context [52]. GATA3 and its paralog GATA2 have a redundant function in establishing the TE lineage in the mouse, as only the double KO and not the single KO mice fail at the preimplantation stage [62]. Interestingly, GATA2 is not expressed in the human outer morula cells (as in the mouse) but only later in the TE of both species [52].

In contrast to the mouse morula, where YAP1 together with TEAD4 directly drive expression of *Cdx2* in the outer cells, concomitantly with the establishment of TE, [50], human embryos show expression of CDX2 only at the blastocyst stage. While in the mouse, the expression of CDX2 and OCT4 become mutually exclusive before the early blastocyst stage [50, 52], in the human, CDX2 is co-expressed with OCT4 in the TE of the early blastocyst and becomes exclusively expressed only in the TE of the late blastocyst [63, 64]. Similar to OCT4, SOX2 is vital for mouse preimplantation development and becomes restricted in expression to the inner cells of the morula. In humans, SOX2 is initially expressed in all nuclei up to the formation of the early blastocyst and becomes confined to the ICM in the expanding blastocyst [52]. There are other prominent differences in TF expression patterns between the mouse and human TE: EOMES, TFAP2C and ID2 are restricted to the mouse TE. However, they are either absent (EOMES) or unrestricted (TFAP2C, ID2) in the human blastocyst [64].

Collectively, despite the evolutionary conservation of blastocyst formation and the first lineage specifications, important molecular differences in timing and expression and thus regulation of the process exist in mouse and human embryos.

#### TFs operating in CTB

**Fig. 4:**
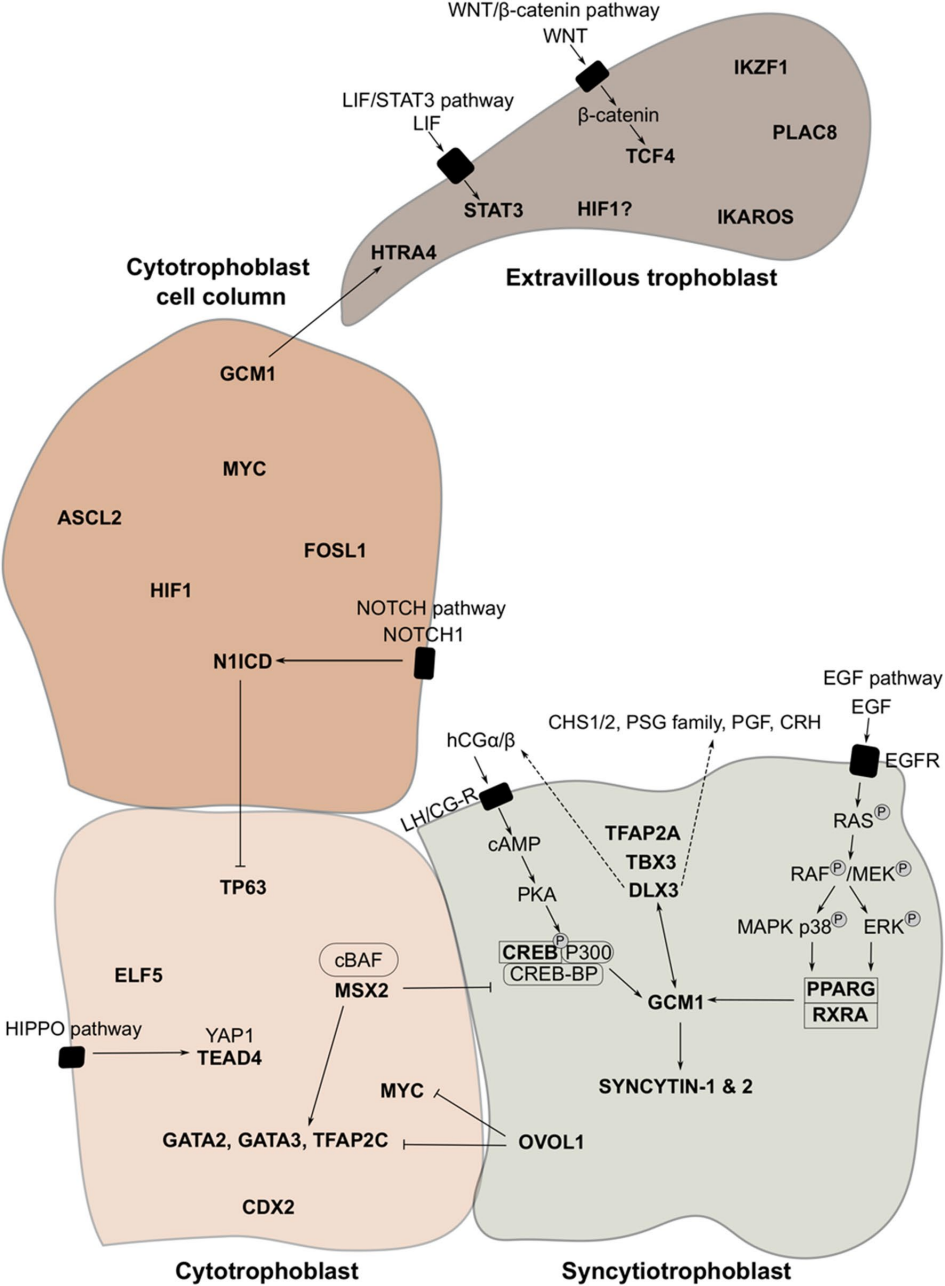
Overview of TFs operating in different types of human trophoblast. TFs are shown in bold. Arrows indicate interactions between TFs, signaling ligands and receptors. Dashed arrows indicate secreted components. The bar line indicates an inhibitory relationship between TFs

The molecular mechanisms, particularly the TFs, driving placental development between the blastocyst implantation and the establishment of the villi are poorly understood. Because researchers have restricted access to human blastocysts and first trimester placental tissue and their potential for genetic studies is very limited, the developmental period of TE-CTB transition remains largely unexplored. However, there is a considerable amount of data on the TFs operating in the first-trimester trophoblast (Fig. 4). Depending on the location, the CTB is the progenitor population for STB and EVT lineages and the TFs controlling its identity are intensely studied. Notably, the recent establishment of hTSCs representing CTB provided an excellent, genetically amenable model that will further propel our understanding of these TF networks [11].

Many TFs present in TE continue to be expressed in CTB, including GATA2/3, TEAD4, and TFAP2C. Recent studies have shown that both in human and mouse, TEAD4 promotes TSC self-renewal and stemness by driving the expression of cell cycle genes and prevents STB and invasive EVT differentiation by silencing differentiation markers [14]. The importance of TEAD4 for multipotency was further corroborated by the finding that idiopathic recurrent pregnancy failures were associated with loss of TEAD4 expression in trophoblast progenitors [14]. Similarly, depletion of YAP1, a cofactor of TEAD4, revealed its involvement in driving self-renewal, proliferation and stemness by transcriptional regulation of cell cycle- and STB-related genes [51]. In summary, the HIPPO pathway controls the trophoblast progenitors in vivo and stem cells in vitro at early (TE) and later (CTB) stages of human placental development, highlighting its universal role in maintaining trophoblast multipotency.

As already mentioned, GATA3, GATA2 and TFAP2C are other conserved TFs, readily expressed in TE, CTB and hTSCs. While the molecular mechanisms underlying the GATA2/3 and TFAP2C action in CTB and hTSCs await elucidation, the hESC-based trophoblast differentiation model has already provided some insights. Even though disputed by some [65], this in vitro system was used to demonstrate that the network of TFs GATA2, GATA3, TFAP2A and TFAP2C regulates trophoblast identity and facilitates the exit from pluripotency, and depletion of GATA3 prevents specification of trophoblast identity [66]. The critical roles of GATA2, GATA3 and TFAP2C in the human CTB and hTSCs are expected, as their depletion in the mouse system resulted in impaired trophoblast development resulting in embryonic lethality [62, 67]. Moreover, GATA2 and TFAP2C gained further prominence, as depletion of either impaired reprogramming of fibroblasts to naïve hESCs, which have the potential to adopt the hTSC state in suitable media conditions [68]. In addition, GATA2, GATA3 and TFAP2C act as powerful, pioneering TFs, i.e., they can bind nucleosomal DNA and nucleate the formation of a new enhancer and hence drive cell fate decisions in diverse developmental contexts [69, 70]. CDX2 is another TF present in both human and murine TE. However, in contrast to the mouse, CDX2 expression is gradually lost during the first trimester of human placental development and is absent in hTSCs [11, 71]. These observations raise questions to what extent CDX2 is an actual human trophoblast marker, whether several progenitor trophoblast subpopulations exist during the first trimester, and whether the hTSCs media lacks the necessary signaling inputs to maintain CDX2 expression.

As previously discussed, the TE in humans gives rise to the proliferative progenitor population of CTB. Interestingly, a number of TFs vital for sustaining the progenitor identity are continuously expressed starting in the TE (e.g., GATA2/3, TEAD4, TFAP2C, etc.). In contrast, others commence their expression only in the CTB and ExE. For instance, expression of ELF5 is conserved in human and mouse trophoblast progenitor populations (CTB and ExE, respectively) as well as in mTSCs and hTSCs, and downregulated upon differentiation. Similarly, epigenetic regulation of the *Elf5/ELF5* promoter by DNA methylation is preserved between these two species [72, 73]. In mice, ELF5 cooperates with EOMES and TFAP2C and acts as a molecular switch governing the balance between mTSCs proliferation and differentiation [37]. ELF5 is essential for mouse placental development and the maintenance of mTSCs; however, its function in the human trophoblast remains to be elucidated [37, 74].

ESRRB, EOMES and SOX2 are indispensable for mouse placental development and maintenance of mTSCs [75–78]. They are lowly expressed (ESRRB and SOX2) or absent (EOMES) from both the CTB and hTSCs [11, 71], further highlighting the species-specific differences during post-implantation trophoblast development. Interestingly, we have recently identified MSX2 as a novel and human-specific regulator of trophoblast identity [15]. While MSX2 depletion results in loss of hTSC self-renewal and spontaneous STB differentiation, MSX2 forced expression blocks it. Mechanistically, MSX2 cooperates with the chromatin-remodeling cBAF complex to prevent premature syncytiotrophoblast differentiation and reinforce the stemness of hTSCs. Hence, MSX2 was established as a repressor of the STB lineage, playing a pivotal role in cell fate decisions governing human placental development and disease. Similar to MSX2, TP63 seems to be specific to the human trophoblast. TP63 is commonly expressed in the basal layer of stratified epithelia, where it reinforces self-renewal and restricts premature differentiation [79]. In the trophoblast context, it is highly expressed in CTB and hTSCs, where it drives proliferation and prevents epithelial-to-mesenchymal transition and differentiation [80].

Taken together, several TFs have been identified in human CTB that maintain the progenitor state and prevent premature differentiation; however, the relationships between these factors in a regulatory network are unclear; some are yet likely to be discovered, and their function may differ from that in mice.

#### TFs operating in STB

As in other cell types, the unique combination of TFs determines the identity of STB Fig. 4). There are two types of human STB: the primitive STB mediates embryonic implantation, and the definitive STB lines chorionic villi from the third week onwards. The exact relationship between these two subtypes is unclear. The villous STB remains in direct contact with maternal blood and ensures optimal pregnancy adaptation. It enables efficient transport of nutrients, gases and metabolites via an enlarged cell surface by microvilli, numerous channels and transporters. It synthesizes and secretes a variety of pregnancy hormones, including human chorionic gonadotropin (hCGA and hCGB), placental lactogen (CSH1), pregnancy-specific glycoproteins (PSGs), placental growth factor (PGF), corticotropin-releasing hormone (CRH) and others [81, 82]. Importantly, STB is devoid of human leukocyte antigen class I (HLA-I) molecules, making it invisible to the potentially reactive T cells [83]. Thus, STB is vital for a functional placenta and successful pregnancy. STB undergoes a tightly regulated turnover and is replenished by the coordinated differentiation and fusion of the underlying CTB. This process is tightly controlled and involves biochemical as well as morphological changes. While the direct signal remains elusive, a prerequisite for differentiation is an exit from the cell cycle [84], followed by repression of genes associated with the progenitor state and activation of genes related to STB function, i.e., involved in nutrient transport, hormone synthesis, and immunomodulation. This differentiation process is coordinated by a subset of TFs and signaling inputs. For instance, hCG promotes syncytialization [85]. Initially, it is secreted by CTB and is thought to induce the formation of the primitive syncytium. During later stages, it is produced by STB and acts as a positive regulator of the syncytialization process. Mechanistically, hCG binding to the luteinizing hormone/choriogonadotropin receptor (LH/CG-R) induces high levels of cAMP and activation of protein kinase A (PKA). In turn, PKA phosphorylates the cAMP response element-binding (CREB) TF, which, together with the CREB-binding protein and histone acetyltransferase P300, promotes expression of fusogenic genes and GCM1 [86–88]. GCM1 is of particular importance as it directly drives expression of the critical fusogenic proteins SYNCYTIN-1 and -2, encoded by the human endogenous retroviruses (HERV) *HERV-W* and *HERV-FRD* [84, 89–91]. *SYNCYTIN*-*1* and *-2* represent domesticated versions of an *env* gene that in viruses encode the envelope glycoprotein mediating infection competency and virus-host membrane fusion. SYNCYTIN-1 and -2 interact with their respective receptors, the sodium-dependent neutral amino acid transporters (ASCT1 and ASCT2) and major facilitator superfamily domain-containing 2a (MFSD2a), respectively. The receptors are localized on the target-cell membrane and induce the cell fusion process that is vital for STB formation [88]. Interestingly, the role of GCM1 in STB morphogenesis is conserved in the murine placenta, as embryos deficient for *Gcm1* show a block of branching morphogenesis and lack of STB [92].

Another conserved TF implicated in human STB formation is the peroxisome proliferator-activated receptor gamma (PPARG). Its expression is modulated by EGF signaling via phosphorylation of P38. PPARG forms a heterodimer with retinoid X receptor alpha (RXRA) and promotes syncytialization by driving transcription of STB-related genes, including GCM1 and SYNCYTIN-1 [93, 94]. Accordingly, mTSCs deficient for *Pparg* fail to differentiate to STB [95], and *Pparg* KO embryos result in embryonic lethality due to a placental phenotype [96]. The TF distal-less 3 (DLX3) has also been demonstrated to control STB identity. Mouse embryos deficient for *Dlx3* show embryonic lethality around E10 and their placentas feature malformations in the labyrinth and spongiotrophoblast compartments [97]. In the human placenta, DLX3 appears to bind and regulate the expression of *CSH1*, *CGA*, *HSD3B1*, and *PGF* and thus regulates STB differentiation [98]. Additionally, it has been demonstrated that DLX3 physically interacts with GCM1 and inhibits its transactivation activity at the *PGF* promoter, among others [99]. These findings provide an example of how an interaction between TFs impacts their activity and outcome. The transcription factor activator protein-2 alpha (AP-2α; TFAP2A) was reported as a critical regulator of biochemical but not morphological differentiation of the human STB [100]. While expression of a dominant-negative version of TFAP2A significantly inhibited induction of vital STB markers, (including hCG, PSG family of genes, PGF, and cytochrome P-450 (CYP11A1)), it did not affect cell fusion, demonstrating the uncoupling of the biochemical and morphological stages of STB differentiation [100]. Interestingly, transcription of *TFAP2A* is regulated by a nuclear receptor subfamily 2, group F, member 2 (NR2F2). Induction of NR2F2 promoted expression of TFAP2A, and conversely, NR2F2 depletion reduced it, leading to impaired STB differentiation [101]. Another TF that is indirectly involved in regulating the STB state is OVOL1. OVOL1 is thought to suppress CTB genes including *MYC*, *ID1*, *TP63* and *ASCL2* to facilitate STB differentiation. Consequently, disruption of *OVOL1* resulted in incomplete silencing of these genes and impaired STB differentiation [102]. Recently, a single cell (sc)RNA-seq analysis on human embryos cultured using a peri-implantation in vitro model identified TBX3 as a novel regulator of STB cell fate [103]. Functional validation experiments have demonstrated that depletion of TBX3 in the JEG-3 cell line blocked STB differentiation [103].

Overall, several TFs have been identified to control STB fate and function; however, their STB context-specific genome-wide binding profiles, interacting proteins, TF networks and transcriptional co-factors remain largely unexplored.

#### TFs operating in EVTs

As stated earlier, the EVT encompasses both the proliferative EVT of stratified cell columns and the invasive EVT. The latter include the eEVT that lines the spiral arteries and the iEVT that migrates through the uterine stroma. The diverse EVT subtypes have unique properties as they perform highly specialized functions and are thus regulated by a subtype-specific set of TFs (Fig. 4). These TFs can be divided into those that promote EVT proliferation and those that promote EVT cell cycle exit, differentiation and invasiveness [104]. Hypoxia has been suggested to promote proliferation of EVT via the hypoxia inducible factor 1 (HIF1) TF, and to inhibit their differentiation into the invasive subtypes, supporting placental growth [105]. However, other studies found the opposite effect: that the hypoxia-HIF pathway stimulates EVT invasiveness [106].

One of the TFs implicated in regulating the EVT identity is achaete-scute family bHLH transcription factor 2 (ASCL2). Mice deficient for *Ascl2* are embryonic lethal due to impaired spongiotrophoblast development and an overabundance of giant cells [107, 108]. In the human placenta, ASCL2 is expressed in CTB and proliferative EVT but not in STB and invasive EVT. Recent reports have shown that disruption of *ASCL2* impaired EVT differentiation of hTSCs and instead induced STB identity [16]. These observations indicate that ASCL2 acts to promote proliferation and inhibits differentiation. Similarly, the FOS like 1 (FOSL1) and MYC TFs are also expressed in CTB and proliferative EVT cell columns but not in the more differentiated lineages. In agreement with this, it has been demonstrated that depletion of FOSL1 in an immortalized EVT cell line HTR-8/SVneo results in loss of proliferation and gain of invasiveness, indicating that its primary role is also to reinforce the undifferentiated, proliferative state [109]. Interestingly, both MYC and FOSL1 are also required for placental development in mice [110, 111].

While GCM1 is a master regulator of STB formation, it is also involved in the regulation of the EVT cell fate. In this context, GCM1 drives the expression of genes promoting invasion, including *HTRA4*, a serine protease that facilitates fibronectin cleavage [112]. Accordingly, expression of GCM1 provokes invasiveness of the BeWo cell line, while depletion of GCM1 in explant cultures reduces it [91]. Thus, GCM1 promotes cell cycle exit and context-dependent differentiation of both STB and EVT.

One of the few known TFs specifically expressed in the iEVT and required for differentiation is placenta-specific protein 8 (PLAC8). While depletion of PLAC8 in explant culture and HTR8/SVneo cell line abrogated EVT migration and invasion, ectopic expression of PLAC8 promoted it [113]. These observations indicate that PLAC8 may play an important role in human placental development and disease as a critical regulator of iEVT invasion and migration. In the mouse placenta, *Plac8* mRNA localizes to trophoblast giant cells at E6.5 and E8.5, and to spongiotrophoblast at E10.5 and E18.5, suggesting a role for PLAC8 in murine placental development [114]. However, a placental phenotype has not been reported in embryos deficient for *Plac8* [115]. Another TF expressed in EVTs is the IKZF1 Ikaros family zinc finger protein 1. The expression of dominant-negative IKZF1 abrogated migration and invasion in an immortalized EVT cell line and IKAROS was demonstrated to promote EVT differentiation [116, 117].

WNT signaling is vital not only for the reinforcement of the progenitor state in CTBs but also plays a role in the determination of EVT identity. Canonical WNT signaling operates via the transcriptional co-activator β-CATENIN that translocates to the nucleus, where it associates with the T-cell factor (TCF)/lymphoid enhancer factor (LEF) family of TFs, driving transcription of diverse genes. It has been demonstrated that WNT3A stimulated EVT migration and invasion, while the addition of the WNT signaling inhibitor DKK-1 blocked it [118]. Similarly, depletion of TCF4 in both villous explant cultures and the SGHPL-5 cell line impaired their migratory and invasive potential [119]. Collectively, these findings indicate that WNT/TCF4 signaling promotes EVT motility and invasiveness. WNT signaling is also vital for the development of the murine placenta, as mice deficient for components and mediators of this pathway show defects in labyrinth formation (WNT2, WNT7B and R-SPONDIN, FZD5), impaired chorioallantoic fusion (*Tcf1/Lef1* dKO), and faulty STB formation (*Bcl9L*) [120–124]. In addition, WNT/β-CATENIN signaling has been demonstrated to activate the expression of *Gcm1* and promote STB-II cell fate [125]. Thus, while indispensable for mouse and human trophoblast development and function, WNT/β-CATENIN signaling plays context-dependent roles. In humans, it sustains CTB progenitor identity and controls migration and invasiveness of the EVT, whereas, in mice, it drives STB identity and labyrinth formation.

Two other pathways that regulate EVT identity are LIF/STAT3 and NOTCH signaling. Signal transducer and activator of transcription 3 (STAT3) is vital for ESC self-renewal as the critical mediator of the LIF/STAT3 signaling. Upon phosphorylation, it undergoes homo- or heterodimerization and translocates to the nucleus, where it regulates the expression of a plethora of genes. Experiments in the choriocarcinoma JEG-3 cell line showed that LIF/STAT3 signaling promotes cell proliferation and invasiveness [126]. Moreover, the STAT3 DNA-binding activity was increased in invasive cells, including first-trimester trophoblast, collectively suggesting that LIF/STAT3 may regulate EVT invasive properties. This notion is in agreement with observations made in murine placentas, where the LIF/STAT3 and the suppressor of cytokine signaling 3 (SOCS3) are implicated in the regulation of placental development [127]. NOTCH1 is expressed at the base of the proliferative cell columns and upon gamma-secretase cleavage gives rise to the NOTCH1 intracellular domain (N1ICD) that acts as a transcriptional activator. Manipulation of NOTCH1 in primary trophoblast models revealed that N1ICD promoted trophoblast survival and repressed CTB markers including *TEAD4* and *TP63* and induced the EVT progenitor-specific genes *MYC* and *VE-CADHERIN* [128]. These findings place NOTCH1 as a key regulator promoting the development of EVT progenitors in the human placenta.

Taken together, although a number of TFs and signaling pathways have been implicated in EVT specification, differentiation and migration, it is likely that more remain to be discovered. Importantly, it is unclear how these TFs exert their function within context-dependent TF networks and with transcriptional co-factors in the EVT lineage.

## Current limitations and outlook

Optimal placental development and function are vital for a successful pregnancy outcome and the wellbeing of the embryo and the mother. These processes require coordinated and dynamic actions of TFs that, in concert with signaling inputs, determine cell identity of specialized trophoblast cell types. Despite their indisputable importance, the molecular mechanisms underlying TF actions in the human trophoblast remain poorly understood. In addition, our knowledge is based on studies performed mainly in transformed trophoblast cell lines and explant cultures, encouraging caution in data interpretation and drawing of conclusion. It will be of great interest to validate these TF studies in more physiologically relevant systems like hTSCs and TOs. The recent establishment of the chemically-defined conditions to culture hTSCs and TOs has been a significant breakthrough, transforming the human trophoblast field. It provided researchers with reliable, well-characterized, and physiologically relevant in vitro models of the human trophoblast. Importantly, these models are scalable, amenable to genetic manipulations and screens, and compatible with various -omics approaches, and thus offer an excellent system to study TFs in the human trophoblast. Indeed, several important findings about TFs have recently been made using this system [14–16].

While hTSCs and TOs provide invaluable tools to study the human trophoblast and TF regulation, researchers should also consider particularities of these models, when interpreting results. For instance, in comparable culture conditions (EGF, CH99021, A83-01, ROCKi), TOs consist of co-existing CTB-like and STB-like populations, while hTSCs remain CTB-like and do not undergo spontaneous STB differentiation. STB differentiation of hTSCs requires the strong inducer forskolin instead. These observations may suggest that TOs constitute a better model of chorionic villi as they recapitulate the co-existence of CTB and STB in a dynamic balance. Indeed, recent findings confirm that TOs represent villous CTB and STB, while hTSCs resemble cells at the base of the cell columns from where EVT derives [129]. Interestingly, EVT differentiation of both hTSC and TOs requires specific growth factor stimuli and seems less robust compared to STB differentiation. These observations may suggest that the commonly used hTSC/TO culture conditions introduce a bias favoring the villous CTB/STB identity. An alternative explanation may point toward inefficient EVT differentiation protocols and intensive efforts are made toward their further optimization (Sandra Haider et al., personal communication). In summary, while hTSCs and TOs offer valuable tools to investigate the role of TFs in the human trophoblast, additional studies are required to bettter characterize these systems better and harness their full potential.

Discoveries made in mESCs, hESCs, and in mTSCs proved transformative to our understanding of the TF-driven molecular processes that determine cell identity and control development. Based on this blueprint, future research efforts in the context of the human trophoblast should focus on identifying novel TFs and their functional characterization, elucidating the cooperation between these TFs, chromatin remodelers and modifiers, and decoding the TF networks that control human trophoblast lineage specification. Particularly important for dissecting the role of trophoblast TFs in the future will be the unbiased study of global effects in gain and loss of function experiments since most studies in the past focused on selected marker genes. Similarly, the genome-wide determination of TF-bound regions and the identification of the TF protein interactomes will provide a holistic comprehension of trophoblast network control, instead of the insular focus on single factors. Finally, the growing human trophoblast expression atlas comprising bulk and single-cell transcriptome datasets will be a crucial resource for benchmarking mechanistic in vitro studies [11, 130–132].

Overall, the field can look forward to an exciting next decade where a combination of new models and technologies will enable breakthroughs in understanding TF control of human placental development and disease.

## Data Availability

Not applicable.
